# Anterolateral minithoracotomy versus median sternotomy for the treatment of congenital heart defects: a meta-analysis and systematic review

**DOI:** 10.1186/1749-8090-7-43

**Published:** 2012-05-04

**Authors:** Chao Ding, Chunmao Wang, Aiqiang Dong, Minjian Kong, Daming Jiang, Kaiyu Tao, Zhonghua Shen

**Affiliations:** 1 Department of Cardiothoracic Surgery, Second Affiliated Hospital, School of Medicine, Zhejiang University, Hangzhou, 310009, PR China

**Keywords:** Minimally invasive surgical procedures, Sternotomy, Congenital heart defect

## Abstract

**Background:**

Anterolateral Minithoracotomy (ALMT) for the radical correction of Congenital Heart Defects is an alternative to Median Sternotomy (MS) due to reduce operative trauma accelerating recovery and yield a better cosmetic outcome after surgery. Our purpose is to conduct whether ALMT would bring more short-term benefits to patients than conventional Median Sternotomy by using a meta-analysis of case–control study in the published English Journal.

**Methods:**

6 case control studies published in English from 1997 to 2011 were identified and synthesized to compare the short-term postoperative outcomes between ALMT and MS. These outcomes were cardiopulmonary bypass time, aortic cross-clamp time, intubation time, intensive care unit stay time, and postoperative hospital stay time.

**Results:**

ALMT had significantly longer cardiopulmonary bypass times (8.00 min more, 95% CI 0.36 to 15.64 min, p = 0.04). Some evidence proved that aortic cross-clamp time of ALMT was longer, yet not significantly (2.38 min more, 95% CI −0.15 to 4.91 min, p = 0.06). In addition, ALMT had significantly shorter intubation time (1.66 hrs less, 95% CI −3.05 to −0.27 hrs, p = 0.02). Postoperative hospital stay time was significantly shorter with ALMT (1.52 days less, 95% CI −2.71 to −0.33 days, p = 0.01). Some evidence suggested a reduction in ICU stay time in the ALMT group. However, this did not prove to be statistically significant (0.88 days less, 95% CI −0.81 to 0.04 days, p = 0.08).

**Conclusion:**

ALMT can bring more benefits to patients with Congenital Heart Defects by reducing intubation time and postoperative hospital stay time, though ALMT has longer CPB time and aortic cross-clamp time.

## Background

The Anterolateral Minithoracotomy (ALMT) is a cardiovascular surgery technique in the purpose of reducing the surgical trauma so that to accelerate recovery and promote the cosmetic outcome, especially for the young female [1]. There have been numerous studies on this subject. Most try to find out that ALMT brings more short-term benefits to patients than MS, such as intubation time, ICU stay time and postoperative hospital stay time [2-7]. Some illustrate the long-term outcome measures in the ALMT group comparing with the MS group [8]. Some introduce their long-term experience on ALMT but do not set the control group [9-17]; others were ambiguous. However, there are some studies complaining about the lung injury resulted from one-lung ventilation applied in ALMT [18-20]. No meta-analysis has been done on ALMT before. Our purpose is to conduct whether ALMT would bring more short-term benefits to patients than conventional MS.

## Methods

### Search strategy

Search for all relevant published articles in English was performed in GOOGLE SCHOLAR, MEDLINE, CENTRAL and EMBASE databases starting from 1997. We assessed the eligibility of every study by more than one author during the search. Our searching keywords were Anterolateral Minithoracotomy, Median Sternotomy, Congenital Heart Defect, Septal Defect, Tetralogy of Fallot and Patent Ductus Arteriosus. Reference lists of every relevant article were searched as well. Because of a lack of randomized controlled trials in this subject, case–control studies were included as an alternate.

### Study selection criteria

We selected the studies according to the following inclusion criteria: (1) the type of studies: RCT should be firstly considered. However, we found a lack of RCT or other prospective studies in ALMT studies. Case–control studies were selected instead; (2) participants: children and adult patients with Congenital Heart Defects undergoing ALMT. The exclusion criteria were: (1) any other type of minimally invasive surgeries; (2) the language of the article was not English; (3) the study did not set a control group, or the control group was not MS.

### Outcome measures

Our outcome measures included cardiopulmonary bypass time, aortic cross-clamp time, intubation time, ICU stay time and postoperative hospital stay time.

### Meta-Analysis

Review Manager (RevMan) V.5.0 was used for statistical analysis. Because the data was continuous, mean differences were measured. We tested heterogeneity by using the χ² test, I² test and degrees of freedom, and we chose to use the random effects model presuming that outcome measures of each study were variable. In this meta-analysis, the risk of bias was not assessed.

### Surgical technique

Two-lumen endotracheal intubation for one-lung ventilation were performed. The skin incision (4–8 cm in length) in the right anterolateral submammary groove through the 4^th^ intercostal space was performed, with minimal rib spreading.To achieve better operative vision, a soft tissue retractor should be employed and both of the right lung and right lobe of the thymus gland should be retracted posteriorly. The superior caval vein was cannulated percutaneously after anesthesia. The right femoral artery and vein were exposed through a small (about 2–3 cm) inguinal incision and cannulated. After the systemic administration of heparin, 28°C-32°C hypothermic cardiopulmonary bypass (CPB) was instituted. Both caval veins were surrounded with tape for ligation and a transthoracic aortic cross-clamp were prepared through the third intercostal space at the anterior and midaxillary line. Myocardial protection was achieved with antegrade cardioplegic solution infusion through the root cannula at the ascending aorta. The defects were repaired with a patch or direct closure. The cardiac anomalies were corrected in almost the same manner as a median sternotomy.

## Results

**Figure 1:**
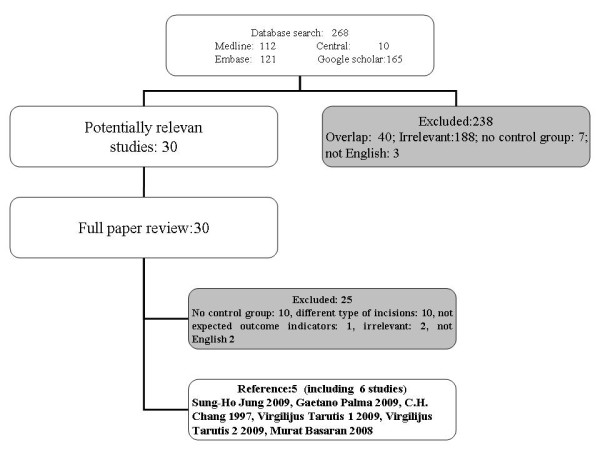
Flow diagram of study inclusion and exclusion criteria: This flow diagram illustrates the databases searched in this review, the resulting number of potential studies subject to our inclusion criteria; and the number and reasons for excluding studies based our exclusion criteria.

Finally, 5 references were selected, including 6 case–control studies, according to our meta-analysis [Table 1[2-6]. One reference [5] included two separate studies called Virgilijus Tarutis 1 2009 (124 patients with Atrial Septal Defect), and Virgilijus Tarutis 2 2009 (70 patients with Ventricular Septal Defect). Excluded studies [Figure 1 were either overlapped (n = 40), irrelevant (n = 191), not English (n = 5), different type of incisions (n = 10), not expected outcome measures (n = 1), or no control groups (n = 17). One reference [7] was highly relevant to our study but written in Chinese. At last, we found 30 studies, in which 6 studies met our criteria. 932 patients were included (384 Atrial Septal Defect, 85 Ventricular Septal Defect, 13 Partial Anomalous Pulmonary Venous Connection, 15 Partial Atrioventricular Canal, 3 Tetralogy of Fallot, 3 Cor Triatriatum, 12 Congenital Mitral Valve Defect, 2 Pericardial Cysts, 415 in the control group not mentioned), on operation interventions 263 undergoing ALMT and 669 MS.

**Table 1:** Study characteristics

**Study**	**Virgilijus Tarutis 1 2009**	**Virgilijus Tarutis 2 2009**	**Gaetano Palma 2009**	**Sung-Ho Jung 2009**	**C. H. Chang 1997**	**Murat Basaran 2008**
Methods	case control study	case control study	case control study	case control study	case control study	case control study
No of patients	17 + 107 = 124	11 + 59 = 70	132 + 415 = 547	9 + 8 = 17	60 + 58 = 118	34 + 22 = 56
Mean age (ALMT/MS)	8.8/23.3	7.7/4.3	10.12/9.5	26.4/38.4	18.8/17.3	21.7/18.6
Sex M:F (ALMT/MS)	Not mentioned	Not mentioned	25:107/170:245	1:8/5:3	25:35/23:35	14:20/2:20
Diagnosis	ALMT:ASD (17)	ALMT: VSD (11)	ALMT: CHD (132)	ALMT: VSD (9)	ALMT: ASD (60)	ALMT: ASD (34)
(ALMT/MS)	MS: ASD(107)	MS: VSD (59)	MS: Not mentioned (415)	MS: VSD (8)	MS: not mentioned (58)	MS: ASD (22)
Operation Interventions	Right anterolateral mini-thoracotomies Vs. Median Sternotomy	Right anterolateral mini-thoracotomies Vs. Median Sternotomy	Right anterolateral mini-thoracotomies Vs. Median Sternotomy	Right anterolateral mini-thoracotomies Vs. Median Sternotomy	Right anterolateral mini-thoracotomies Vs. Median Sternotomy	Right anterolateral mini-thoracotomies Vs. Median Sternotomy
Outcome measures	CPB(min)	CPB(min)	CPB(min)	CPB (min)	CPB (min)	CPB (min)
	——	ACCT(min)	ACCT(min)	ACCT(min)	——	ACCT(min)
	Intubation time(h)	Intubation time(h)	Intubation time(h)	Intubation time(h)	Intubation time(h)	Intubation time(h)
	hospital stay(d)	hospital stay(d)	hospital stay(d)	hospital stay(d)	hospital stay(d)	hospital stay(d)
	ICU time (d)	ICU time (d)	——	——	ICU time (d)	ICU time (d)

ACCT, aortic cross-clamp time; ALMT, Anterolateral Minithoracotomy; ASD, Atrial Septal Defect; CHD, Congenital Heart Defect; CPB, cardiopulmonary bypass time; ICU, intensive care unit; MS, Median Sternotomy; VSD, Ventricular Septal Defect.

Table 1 illustrates detail characteristics of these 6 studies. The following results are presented as mean differences in outcome measures between ALMT and MS in the random effects model.

### Cardiopulmonary bypass time (min)

**Figure 2:**
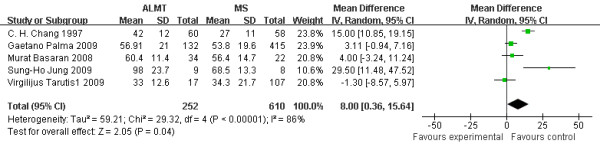
**Cardiopulmonary bypass time (min): the figure illustrates the result of meta-analysis on CPB time.** CPB time was significantly longer in the ALMT group (p = 0.04).

Cardiopulmonary bypass time (CPB time) is a useful operative measure to compare the difficulty among different cardiovascular surgery. The ALMT group had a significant longer CPB time (8.00 min more, 95% CI 0.36 to 15.64 min, p = 0.04). One study (Virgilijus Tarutis 2 2009 [5]) was not included without CPB time [Figure 2.

### Aortic cross-clamp time (min)

**Figure 3:**
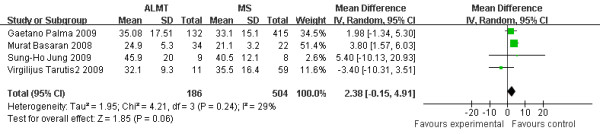
**Aortic cross-clamp time (min): the figure illustrates the result of meta-analysis on aortic cross-clamp time (ACCT).** Four of six studies included proved a longer aortic cross-clamp time in ALMT, but not significant (p = 0.06).

Aortic cross-clamp time (ACCT) can also imply the difficulty of a cardiovascular surgery. There were two studies not mentioning ACCT ( [C. H. Chang 1997 3] and [Virgilijus Tarutis 2 2009 5]). Though other four studies proved a longer aortic cross-clamp time in ALMT, but it was insignificant (2.38 min more, 95% CI −0.15 to 4.91 min, p = 0.06) [Figure 3.

### Intubation time (hrs)

**Figure 4:**
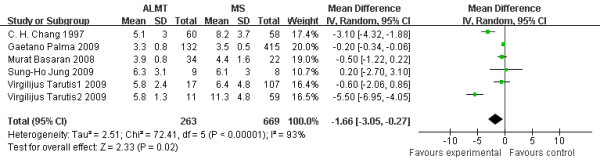
**Intubation time (hrs): the figure illustrates the result of meta-analysis on intubation time.** There was a statistically insignificant reduction in intubation time. This was 1.66 hrs less in the ALMT group (p = 0.02).

Intubation time can represent the degree of impairment of lung function in patients undergoing thoracotomies. There was a statistically significant reduction in intubation time. This was 1.66 hrs less in the ALMT group (95% CI −3.05 to −0.27 hrs; p = 0.02) [Figure 4].

### ICU stay time (days)

**Figure 5:**
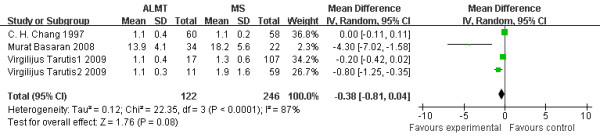
**Intensive care unit stay time (days): the figure illustrates the result of meta-analysis on intensive care unit stay time (ICU).** Only four of six studies presented useable data and included in this analysis. As illustrated in Figure 4, ICU stay time was shortened by 0.38 days in the ALMT group; however, the difference again failed to reach statistically significant levels (p = 0.08).

ICU stay time is a sensitive indicator suggesting the recovery of postoperative patients. As illustrated in Figure 5, ICU stay time was shortened by 0.38 days in the ALMT group; however, the difference again failed to reach statistically significant levels (95% CI −0.81 to 0.04 days; p = 0.08). The [Gaetano Palma 2009 2] and [Sung-Ho Jung 2009 4] were excluded because of a lack of data of ICU stay time [Figure 5.

### Postoperative hospital stay time (days)

**Figure 6:**
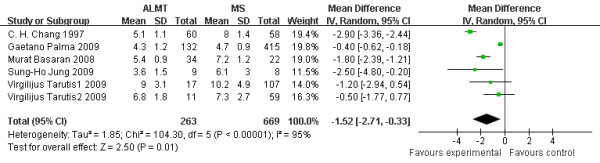
**Postoperative hospital stay time (days): the figure illustrates the result of meta-analysis on postoperative hospital stay time.** The mean difference of all the studies showed that hospital stay time was significantly shortened by 1.52 days in the ALMT group than the MS group (p = 0.01).

Postoperative hospital stay time is another outcome measure demonstrating the recovery of patients after surgery. In Figure 6, the mean difference of all the studies showed that postoperative hospital stay time was significantly shortened by 1.52 days in the ALMT group than the MS group (95% CI −2.71 to −0.33 days; p = 0.01) [Figure 6].

## Discussion

Our meta-analysis was performed to compare the short-term postoperative outcome measures in 6 published studies between Anterolateral Minithoracotomy (ALMT) and Median Sternotomy (MS). The data obtained from the 6 studies was synthesized and a statistical view was drawn on the potential benefits of an ALMT over a full MS for the radical correction of Congenital Heart Defects. The outcome measures were as follow: cardiopulmonary bypass time, aortic cross-clamp time, intubation time, ICU stay time and hospital stay time.

According to our analysis, we found ALMT had a significant longer CPB time (p = 0.04) but a significant shorter time in both intubation time (p = 0.02) and postoperative hospital stay (p = 0.01). There was no previous meta-analysis showing this trend. The reduction in postoperative hospital stay time by 1.52 days (p = 0.01) and ICU stay time by 0.38 days (p = 0.08) can bring potential financial advantages to patients with Congenital Heart Defects.

ALMT uses the technique called “one-lung ventilation” in order to fully expose to the surgery field. Some researches on “one-lung ventilation” pointed out the possibility of lung injury induced by one-lung hyperventilation, stretch of lung and pleural damage [18-20]. If the lung injury happened more severe during ALMT, the intubation time should be longer than MS. However, we found intubation time did not become longer in the ALMT group according to our analysis. It was proved that shorter intubation time by 1.66 hrs (p = 0.02) implied the lung injury would not induce worse short-term outcomes in patients with Congenital Heart Defects undergoing ALMT. More studies should be taken to find out the reasons.

MS has been the conventional approach for the correction of cardiac defects. However, ALMT have been applied more widely in both adult and pediatric populations, especially in females. The incisions of ALMT range 4–8 cm [2-7,21], while those of conventional MS were much longer and more unsightly. One research estimated the satisfactions of the cosmetic result of ALMT, and found 282 (91.5%) of 308 patients were satisfied [1]. Another advantage of ALMT is that it maintains the continuity and integrity of the bony thorax, thereby preventing pectus carinatum.

This study was limited as it only included 6 case–control studies, with variable outcome measures. No randomized controlled trials or other prospective studies were searched out. The risk of bias was not assessed during this meta-analysis. The total number of patients included in this study was 932, but the composition of the sample was complex and farraginous, different from the general population. At the beginning we wanted to limit our study to one single disease. But it was difficult to find enough studies. Another problem was that some studies lacked operative data. There were two studies not mentioning aortic cross-clamp time (C. H. Chang 1997 and Virgilijus Tarutis 2 2009), two studies not mentioning ICU stay time (Gaetano Palma 2009 and Sung-Ho Jung 2009), and one study not mentioning CPB time (Virgilijus Tarutis 2 2009 ). All above would weaken the probative force of our meta-analysis results.

Three meta-analyses have already been performed comparing mini-sternotomy versus conventional sternotomy for aortic valve replacement [1,22,23], including randomized controlled trials and non-randomized studies. They concluded that mini-sternotomy can be performed safely for aortic valve replacement without an increased risk of death or any major complications [22], or a reduction of ICU stay time [23], or with no clinical benefits [1]. On the other hand, we excluded nine studies with no control groups [9-17,21], ranging from 10 to 683 in the sample size. But it was hard to ignore these studies. These studies, with 1642 patients in total, summarized the long-term clinical experience on ALMT useful to make clinic decision and ensure the safety of ALMT.

## Conclusion

ALMT can bring more benefits to patients with Congenital Heart Defects by reducing intubation time and postoperative hospital stay time, though ALMT is a more complex surgery process with longer CPB time and aortic cross-clamp time than MS.

## Abbreviation

ALMT, Anterolateral minithoracotomy; MS, Median sternotomy; CI, Confidence interval; CPB, Cardiopulmonary bypass; ACCT, Aortic cross-clamp time; ICU, intensive care unit; ASD, Atrial septal defect; VSD, Ventricular septal defect; CHD, Congenital heart defect; RCT, Randomized controlled trial; min, Minutes; hrs, Hours.

## Competing interests

The authors declare that they have no competing interests.

## Authors’ contributions

CD and ZHS carried out to study design. CD and CMW carried out data analysis and manuscript writing. ZHS, AQD, MJK, DMJ and JFQ participated in writing the manuscript. All authors read and approved the final manuscript.
